# Design of Conductive Hydrogels Based on the Synergistic Effects of Hydrophobic Frameworks and Dual Antifreeze Strategies, Suitable for Wearable Flexible Sensors

**DOI:** 10.3390/polym18111299

**Published:** 2026-05-25

**Authors:** Jijun Luo, Sainan Wang, Xiangtong Jian, Kenan Yang, Bin Du, Mengwei Yin, Shisheng Zhou

**Affiliations:** 1 School of Mechanical Engineering, Xi’an University of Technology, Xi’an 710054, China; 2 Shaanxi Energy Institute, Xianyang 712000, China; 3 Shaanxi Collaborative Innovation Center of Green Intelligent Printing and Packaging, Xi’an University of Technology, Xi’an 710054, China; 4 Faculty of Printing, Packaging Engineering and Digital Media Technology, Xi’an University of Technology, Xi’an 710048, China

**Keywords:** anti-freezing, conductive hydrogel, phytic acid, flexible sensor

## Abstract

This study focused on a three-dimensional cross-linked hydrophobic association (PS) hydrogel framework. Phytic acid (PA) was selected as both a dopant and an antifreeze agent, and it was combined with an ethylene glycol/water binary solvent to construct a dual antifreeze system. The resulting composite conductive hydrogel, E/PS/PA-PPy, exhibited synergistically enhanced electrical conductivity, mechanical strength, and antifreeze properties. At a PA concentration of 0.1 M, a structurally uniform and ordered three-dimensional network was formed. The PS/PA-PPy hydrogel exhibited an elongation at break of 2595.7% and a high conductivity of 1.8 S/m, while maintaining excellent flexibility and adhesion. Owing to the synergistic antifreeze effect, the freezing point of the E/PS/PA-PPy hydrogel was reduced to −42.3 °C, and after 35 days of room-temperature storage, the weight loss was less than 7%, indicating outstanding water retention. The assembled flexible strain sensor exhibited a sensitivity of 2.09, with response and recovery times both less than 0.25 s. Notably, it exhibited good cyclic stability and accurately monitored human movements. Furthermore, the sensing performance remained stable without significant attenuation even at −20 °C. The results demonstrate the broad application prospects of the hydrogel in flexible electronics such as wearable health monitoring systems and human–machine interfaces in extreme environments.

## 1. Introduction

In recent years, conductive hydrogels—three-dimensional flexible materials formed through the cross-linking of hydrophilic polymers—have garnered significant attention [1,2,3]. Due to their unique structures and multifunctional properties, these hydrogels have been widely applied in the field of flexible electronics [4,5], including human–machine interfaces [6,7], electronic skin [8], and triboelectric nanogenerators [9,10]. However, conductive hydrogels generally have a high water content, making them prone to freezing in low-temperature environments. This freezing disrupts their internal conductive pathways, leading to a complete loss of their key properties such as their electrical conductivity, elasticity, and adhesion [11,12]. As a result, the application of the hydrogels in extreme outdoor environments with low temperatures is significantly limited [13]. The simultaneous optimization of the mechanical strength, adhesion, antifreeze properties, and electrical conductivity of hydrogels is challenging. Therefore, developing conductive hydrogels that integrate multiple key functionalities holds significant scientific and practical importance.

Conductive polymers [such as polyaniline, polypyrrole (PPy), and polythiophene] are typically used to construct hydrogels due to their notable advantages including their low cost, low density, ease of processing, and tunable functionality [14,15,16,17,18]. The electrical conductivity of conducting polymers is primarily determined by their doping structure, which is commonly modified through chemical or electrochemical doping to introduce charge carriers and optimize the molecular chain arrangement, thereby enhancing conductivity [19,20]. Compared to inorganic small-molecule acids, macromolecular protonic acids possess multifunctional structures that enable stable doping through multi-point ionic bonds and hydrogen bonds. At the same time, they utilize steric hindrance to suppress agglomeration, construct porous networks, and increase active sites. They also promote charge delocalization along the polymer chains, thereby enhancing electrical conductivity [21,22,23]. This effectively addresses the shortcomings of inorganic acids—namely, their weak binding strength and tendency to aggregate—resulting in superior overall doping efficiency and comprehensive performance. Phytic acid (PA) is a non-toxic, naturally occurring organic phosphorus compound characterized by a large number of phosphate groups within its molecular structure, giving it excellent doping properties [24]. It can serve as an efficient proton-doping agent for conductive polymers, as well as a cross-linking agent to enhance their structural density, thereby improving the mechanical properties and electrochemical stability of the polymers [25]. Furthermore, PA molecules possess both hydrogen bond donor and acceptor capabilities, enabling them to form stable hydrogen bonds with water molecules within the hydrogel system. This interaction inhibits the formation and growth of ice crystals at low temperatures, significantly enhancing the freeze–thaw resistance of the hydrogel [26,27,28]. On the basis of the aforementioned characteristics, in this study, a novel antifreeze conductive hydrogel was designed and fabricated to overcome the core issues facing conductive hydrogels: their failure at low temperatures, their weak mechanical properties, and their limited applicability. The synthesized hydrogel featured a phosphoric acid-doped PPy as the conductive core, combining a broad adaptability to temperature with excellent mechanical performance to address the critical challenges typically associated with these materials.

In this study, E/PS/PA-PPy hydrogels were prepared by constructing a hydrophobic associative framework and introducing a synergistic effect between dual freeze resistance and proton doping, thereby overcoming the trade-off between high tensile strength, freeze resistance, conductivity, and adhesion. We synthesized a three-dimensional cross-linked hydrophobic association (PS) hydrogel framework using a thermally initiated free-radical copolymerization process. PA was used as a dopant and an antifreeze agent, in combination with an ethylene glycol (EG)/water binary solvent system, to construct a dual antifreeze system. PA was uniformly doped into the PPy structure, which effectively regulated the formation of a well-organized, uniformly porous three-dimensional network. This conferred the hydrogel with good flexibility, interfacial adhesion, and outstanding electrical conductivity, with its maximum conductivity reaching 1.8 S/m. Leveraging the synergistic antifreeze effects of PA and EG, the freezing point of the gel was lowered to −42.3 °C, enabling it to maintain favorable mechanical stability even at an extremely low-temperature of −25 °C. When assembled into a flexible strain sensor, the hydrogel could accurately detect various human movements, with stable sensing signals and negligible attenuation even at −20 °C. These results demonstrate the potential of the hydrogel for applications in wearable health monitoring, extreme-environment human–machine interfaces, and various flexible electronic devices.

## 2. Experimental Section

### 2.1. Materials

Cetyltrimethylammonium bromide (CTAB), EG, acrylic acid (AA), PA (50% solution), stearyl methacrylate (SMA), ammonium persulfate (APS), and Py were sourced from Shanghai Maclin Biochemical Technology Co., Ltd. (Shanghai, China).

### 2.2. Preparation of Hydrogels

To prepare the PS hydrogel, first, 3 g of CTAB powder was dispersed in 20 mL of deionized water through stirring. Next, 1 g of SMA and 10 g of AA were added sequentially and stirred for 3 h and 1 h, respectively, until the system was homogeneous. Finally, APS was added as a thermal initiator, and the resulting pre-polymer solution was injected into a mold and maintained at 60 °C for 3 h to obtain the PS hydrogel.

To prepare the E/PS/PA-PPy antifreeze hydrogel, first, PA solutions with varying concentrations were prepared using 20 mL deionized water as the solvent. Then, 0.55 mL of Py solution was added to each solution, and the mixture was stirred thoroughly to obtain Py/PA blends of various concentrations. Next, the synthesized PS hydrogel was immersed in a 0.4 mol/L APS solution for 1 h, washed with deionized water to remove surface residues, and dried. The hydrogel was then immersed in the prepared Py/PA mixture at 4 °C for 4 h to obtain the PS/PA_x_-PPy hydrogel. Finally, the hydrogel was transferred into an EG/H_2_O binary solvent and soaked for 1 h to obtain the desired E/PS/PA-PPy antifreeze hydrogel. The detailed compositions of all hydrogels are provided in Table S1.

### 2.3. Characterization

#### 2.3.1. Scanning Electron Microscopy and Energy-Dispersive Spectroscopy Analysis

The microstructure of the antifreeze hydrogel was studied using scanning electron microscopy (SEM, SU-8010, Hitachi, Tokyo, Japan). Moreover, the micro-regional elemental composition and distribution characteristics of the freeze-dried samples were analyzed through energy-dispersive spectroscopy (EDS).

#### 2.3.2. Spectral Analysis

Fourier-transform infrared (FTIR) spectroscopy (IR Spiri, Shimadzu, Kyoto, Japan) was used to characterize the chemical functional groups and structure of the hydrogel samples in the 500–4000 cm^−1^ wavenumber range. Moreover, to analyze the elemental composition and chemical bonding states of the freeze-dried samples, they were cut into appropriate sizes and X-ray photoelectron spectroscopy (XPS) was performed using the Al Kα radiation. Finally, X-ray diffraction (XRD) was performed to investigate the amorphous and crystalline characteristics of the samples.

#### 2.3.3. Mechanical Property Testing

For tensile property tests, 50 × 10 × 1.2 mm hydrogel specimens were used. The tests were conducted at room temperature using an Instron 5500 universal testing machine (Instron, Norwood, MA, USA), with five parallel specimens tested per group. The elastic modulus was calculated based on the slope of the initial linear segment of the stress–strain curve.

#### 2.3.4. Water Retention Test

The water retention capacity of the hydrogel at room temperature was determined using the weight method; the sample was placed in an open weighing dish, and the initial weight (W_0_) and the weight at a specific time (W_t_) were recorded. The water retention rate was calculated using the following formula:

Water retention rate = (W_t_/W_0_) × 100%.

#### 2.3.5. Thermal Properties and Anti-Freezing Ability

Thermogravimetric analysis (TGA) was performed to evaluate the properties thermal, stability, and chemical structure of the hydrogel. The analysis was carried out in a nitrogen atmosphere in the temperature range from 30 to 600 °C at a heating rate of 15 °C per minute. The low-temperature phase transition behavior of the hydrogel was studied using differential scanning calorimetry (DSC). The sample was placed in an aluminum crucible and heated from 25 °C to −80 °C at a rate of 5 °C/min under a nitrogen atmosphere, and the phase transition temperature and heat flow were recorded.

#### 2.3.6. Conductivity Testing

The electrical properties of the samples were investigated using a electrochemical workstation (CHI 660E, CH Instruments, Inc., Shanghai, China). First, the sample was secured horizontally and attached to electrodes. Next, the resistance–time measurement mode was selected, and the resistance value was recorded once the signal stabilized. The conductivity (σ) of the sample was the calculated using the following formula:

$$\sigma =\frac{d}{R\times A}$$

where σ represents the hydrogel conductivity (S/m), R is the sample resistance (Ω), d is the electrode spacing (cm), and A is the sample cross-sectional area (cm^2^).

#### 2.3.7. Sensor Characterization

The strain- and pressure-sensing performance of the hydrogel was studied in both tensile and compression modes. For this, a digital source meter (Keithley 2461, Tektronix/Keithley Instruments, Solon, OH, USA) was used to acquire real-time resistance signals from the hydrogel during deformation in conjunction with an universal testing machine (Instron, 5500, Illinois Tool Works Inc., Norwood, MA, USA).

## 3. Results and Discussion

**Figure 1:**
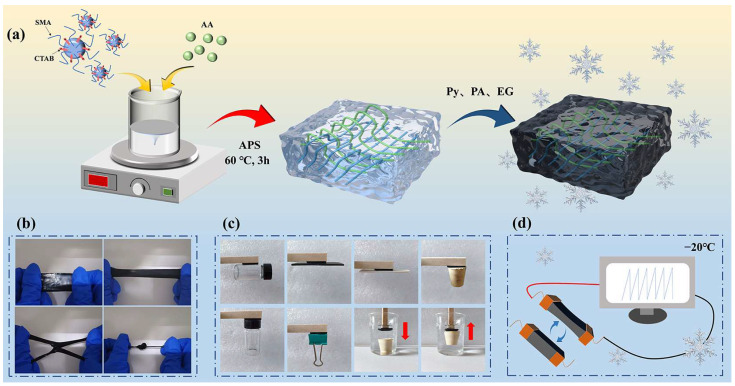
E/PS/PA-PPy freeze-resistant conductive hydrogel. (**a**) Schematic of preparation. (**b**) Macroscopic mechanical property testing. (**c**) Adhesion properties. (**d**) Sensing characteristics.

As shown in Figure 1a, AA and octadecyl methacrylate underwent radical copolymerization to form a three-dimensional cross-linked polymer network, producing a stable PS polymer scaffold. In PS hydrogels, hydrophobic association microdomains acted as energy dissipation centers and flexible network nodes, which greatly enhanced toughness and cyclic stability without sacrificing stretchability. Meanwhile, regulating phase separation formed a uniform porous structure, facilitated the uniform loading of PPy and the construction of conductive pathways. During synthesis, APS (an oxidizing agent) was pre-adsorbed onto the scaffold, which was immersed into the Py/PA mixture, where Py underwent in situ oxidative polymerization at low temperatures to form PPy In this process, PA acted as a dopant; its strong acidity promoted the polymerization of Py and protonated the amino groups of the PPy chains, forming polariton/dipolariton carriers, which significantly enhanced the conductivity of the hydrogel. Furthermore, the abundant P-OH groups in the PA molecules formed strong hydrogen bonds with the C-OH groups in the polyacrylic acid chains, trapping free water molecules and inhibiting ice crystal growth. This provided excellent freeze resistance to the hydrogel. Notably, in this study, synergistic freeze resistance was achieved by immersing the hydrogel in an EG/H_2_O binary solvent, ultimately yielding an E/PS/PA-PPy hydrogel exhibiting both good conductivity and freeze resistance. As shown in Figure 1b–d, the hydrogel exhibited excellent mechanical and adhesive properties, enabling it to stably adhere to objects in an aqueous environment. The results indicate that the hydrogel can be potentially applied in the field of flexible sensors.

**Figure 2:**
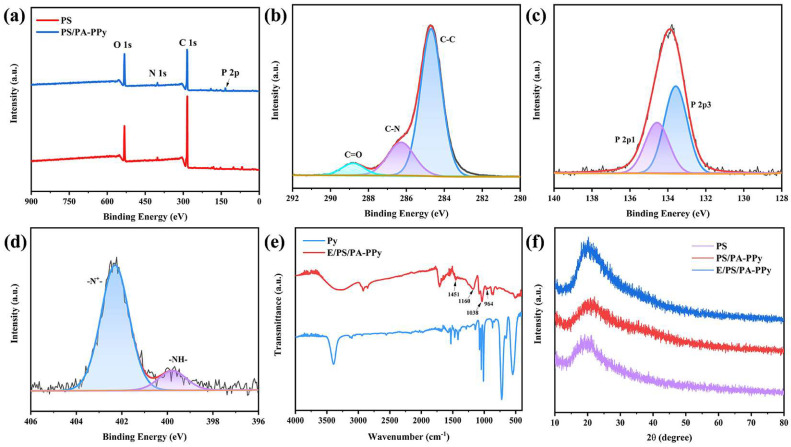
(**a**) Full XPS curves of PS and PS/PA-PPy. (**b**) High-resolution C 1s XPS curve of PS/PA-PPy. (**c**) High-resolution P 2p XPS curve of PS/PA-PPy. (**d**) High-resolution N 1s XPS curve of PS/PA-PPy. (**e**) FTIR spectra of Py and E/PS/PA-PPy. (**f**) XRD patterns of PS, PS/PA-PPy, and E/PS/PA-PPy.

XPS was performed to characterize the chemical state and bonding environment of PS and PS/PA-PPy hydrogels. The total spectra of the two hydrogels are presented in Figure 2a. The PS/PA-PPy hydrogel showed a P 2p peak at 133.5 eV, corresponding to the characteristic binding energy of phosphorus in PA. Figure 2b shows the high-resolution C 1s spectrum of the PS/PA-PPy hydrogel. Peak fitting produced three characteristic peaks at the following energies: 284.8 eV, corresponding to the C-C bond; 286.3 eV corresponding to the C-N or C-O bond; and 288.8 eV corresponding to the C=O bond. Figure 2c shows the high-resolution P 2p spectrum, with characteristic peaks appearing at 133.6 eV (P 2p3/2) and 134.6 eV (P 2p1/2), confirming the doping of PA into the hydrogel. Peak fitting revealed two distinct peaks at 399.8 eV and 402.3 eV, corresponding to -NH- and the positively charged amine group (-N^+^-), respectively. The presence of -N^+^- confirms that PA has been successfully doped into and modified PPy [29]. PA protonates the PPy molecular chains, induces the formation of polaritons and dipolaritons, promotes π-electron delocalization, and improves carrier transport; simultaneously, its polyphosphate groups regulate the ordered arrangement of PPy, suppress aggregation, and construct a continuous conductive network.

Figure 2e shows the FTIR spectra of the Py monomer and the E/PS/PA-PPy hydrogel. As illustrated, after Py was doped with PA and polymerized into the conductive PPy network within the hydrogel matrix, several characteristic absorption peaks of the Py monomer disappeared, while new absorption peaks corresponding to the polymerized and doped PPy structure emerged. The spectrum showed characteristic PPy peaks at 1451 cm^−1^ and 1038 cm^−1^, corresponding to the in-plane bending vibrations of C=C and C-H bonds in the PPy ring, respectively [30]. Moreover, characteristic PPy peaks in the PA-doped state were observed at 1160 cm^−1^ and 964 cm^−1^, corresponding to the P-O and P-O-C stretching vibrations, respectively [31]. These new peaks provide direct evidence that PA has been successfully incorporated into and interacted with the PPy chains.

XRD was utilized to investigate the crystalline structure and molecular chain order of composite hydrogels. As shown in Figure 2f, pure PS hydrogel presents a broad diffuse diffraction peak near 2θ≈20°, demonstrating its typical amorphous structure with disordered molecular chains. After introducing PA-PPy, the PS/PA-PPy composite exhibits nearly the same amorphous region with slightly increased diffraction peak intensity, indicating that the incorporation of PA and PPy does not destroy the inherent network structure of PS hydrogel and effectively improves the molecular chain order of the system. After the incorporation of EG, the diffraction peak intensity was remarkably enhanced, which can be attributed to the fact that EG improved the physical crosslinking density and structural order of the system via hydrogen bond reconstruction [32]. The results reveal that phytic acid can regulate the ordered arrangement and inhibit the aggregation of PPy, and ethylene glycol can further optimize the stacking mode of molecular chains, thus jointly constructing a continuous conductive network.

**Figure 3:**
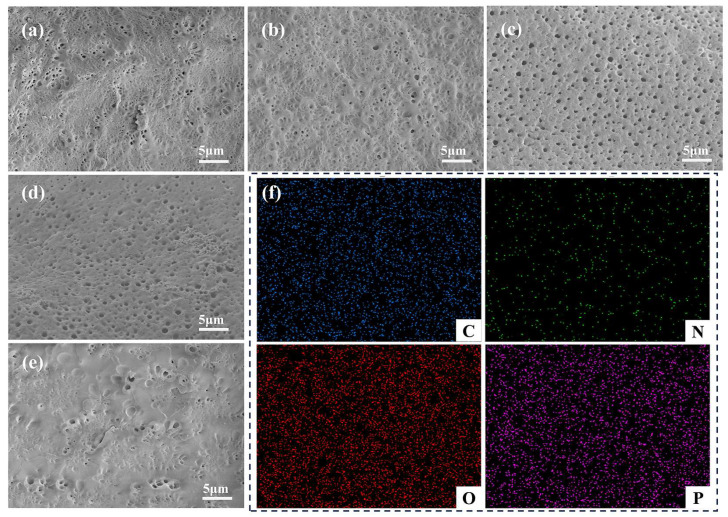
(**a**) SEM image of PS/PPy hydrogel. (**b**–**e**) SEM images of PS/PA-PPy hydrogel samples with different PA concentrations (0.05, 0.1, 0.15, 0.2 M). (**f**) EDS elemental (C, N, O, P) mapping images of PS/PA_0.1_-PPy hydrogel.

To investigate the effect of PA content on the microstructure, SEM was performed on PS/PPy and PS/PA-PPy hydrogels with different PA concentrations. Figure 3a shows an SEM image of the hydrogel without PA doping. As shown, the surface of the hydrogel had an irregular porous structure with pores of varying sizes and a disordered distribution, showing no obvious pattern. Figure 3b shows the SEM image of the PS/PA-PPy hydrogel with a PA concentration of 0.05 M. As can be observed, the pores were finer and more uniformly distributed, indicating that a small amount of PA regulated phase separation, reducing the size of the phase domains. Figure 3c shows an SEM image of the PS/PA-PPy hydrogel with a PA concentration of 0.1 M. The pore uniformity and pore density were optimal, indicating that PA was the most effective at this concentration, making it conducive to the formation of a well-organized three-dimensional network structure. SEM images of the PS/PA-PPy hydrogel with PA concentrations of 0.15 M and 0.2 M are presented in Figure 3d and Figure 3e, respectively. The images showed a slight increase in the pore size, with localized pore coalescence, and large areas of continuous phase, indicating that an excess of PA disrupted the network structure of the hydrogel. The microstructural characterization revealed that the ideal network structure was formed at a PA concentration of 0.1 M. Thus, the uniform incorporation of PA into the three-dimensional network of the hydrogel was verified. For this, EDS was performed on freeze-dried PS/PA_0.1_-PPy antifreeze hydrogel samples to analyze the micro-area elemental composition and distribution. The EDS elemental mapping images (Figure 3f) revealed the uniform distribution of C, N, O, and P within the gel. This confirmed the uniform incorporation of PA into the network structure of the PS/PA-PPy_0.1_ antifreeze gel, as phosphorus originates solely from PA.

**Figure 4:**
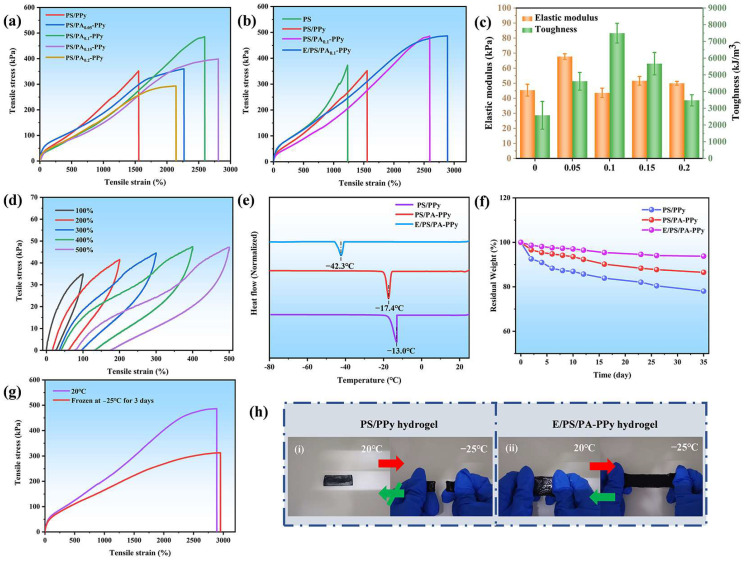
(**a**) Tensile stress–strain curves of PS/PA-PPy hydrogel samples with different PA concentrations. (**b**) Tensile stress–strain curves of various hydrogel samples. (**c**) Changes in elastic modulus and toughness with varying PA content. (**d**) Cyclic tensile curves of E/PS/PA-PPy. (**e**) DSC freezing point test. (**f**) Water retention curves recorded over 35 days. (**g**) Comparison of tensile curves at room temperature and after freezing at −25 °C. (**h**) Photographs demonstrating flexibility at room temperature and −25 °C: (**i**) PS/PPy hydrogel and (**ii**) E/PS/PA-PPy hydrogel.

To investigate the effect of PA concentration on the mechanical properties, tensile tests were performed on the PS/PA-PPy hydrogel samples with different PA concentrations. As shown in Figure 4a,c, with increasing PA concentration, the stress and toughness of the PS/PA-PPy hydrogel first increased and then decreased; the elongation at break rose from 1556.8% to 2595.7%, and then it dropped to 2142.9%. At lower PA concentrations, PPy doping was not effective. As a result, the network structure was relatively loose, failing to form a complete cross-linked network, and stress concentration in the PS/PA-PPy hydrogel resulted in minimal strain. With increasing PA content, a polymer network structure formed in the PS/PA-PPy hydrogel, leading to an increase in elongation at break. However, with a further increase in PA concentration, excessive PA caused extreme segment aggregation locally, while adjacent regions experienced insufficient cross-linking due to limited diffusion. This led to degradation of the three-dimensional network. Notably, the aggregation of conductive material increased the cross-linking density of the hydrogel, restricting the movement of molecular chains and consequently reducing the elongation at break.

The tensile test results of four hydrogels, namely PS, PS/PPy, PS/PA_0.1_-PPy, and E/PS/PA_0.1_-PPy, are displayed in Figure 4b. The neat PS hydrogel possesses a dynamically reversible physical cross-linking network based on hydrophobic association, in which the hydrophobic microdomains serve as energy dissipation centers during deformation, endowing the hydrogel with excellent stretchability and mechanical toughness. Upon the introduction of PPy and PA, PA acts as an effective dopant for PPy. It regulates the internal network structure of the hydrogel through hydrogen bonding, enhances the slippage and extensibility of molecular chains, and thus significantly increases the elongation at break of the hydrogel. The stress response of the E/PS/PA_0.1_-PPy after immersion in an EG/H_2_O binary solvent was further reduced, and its toughness was enhanced. This series of changes clearly demonstrated that PA doping and EG treatment synergistically regulated the mechanical properties of the hydrogel, producing a balance between ductility and stiffness and endowing the material with excellent toughness.

In this study, a dual-pronged antifreeze strategy was used to enhance the low-temperature adaptability of the hydrogel. DSC testing (Figure 4e) validated the effectiveness of the design: the addition of PA lowered the freezing point of the hydrogel to −17.4 °C, which further reduced to −42.3 °C after immersion in the EG/H_2_O binary solvent, demonstrating excellent freeze resistance. The phosphate groups in PA can form a strong network of hydrogen bonds with water molecules. On the one hand, this restricts the movement of water molecules and inhibits their ordered aggregation, thereby preventing ice crystal nucleation; on the other hand, it blocks the bonding of water molecules, hindering ice crystal growth and coarsening. Notably, the EG/H_2_O binary solvent system formed a stable hydrogen bond network, further suppressing ice formation and reducing water loss, thereby maintaining the structure and mechanical properties of the gel. These two components synergistically enhanced freeze resistance by improving the material’s anti-icing and anti-dehydration abilities, broadening the low-temperature application potential of the hydrogel. The TGA curves (Figure S1) showed that PS/PA_0.1_-PPy exhibits a slower thermal degradation rate and higher thermal stability. This indicates that phytic acid significantly enhances the crosslinking density of the three-dimensional network by strengthening hydrogen bonding interactions within the system, thereby endowing the hydrogel with superior thermal stability [33]. Moreover, moisture retention testing (Figure 4f) demonstrated the outstanding water retention capacity of the E/PS/PA-PPy hydrogel, with a weight loss of less than 7% over 35 days. PA doping enhances the hydrogen-bonding crosslinking network within the hydrogel and improves the matrix’s ability to bind water molecules, while the introduction of EG further optimizes the solvation environment of the system, lowers the vapor pressure of water molecules, and suppresses the volatilization of free water. Furthermore, mechanical testing at low temperatures validated the potential of the hydrogel in real-world environments. Notably, after being exposed to −25 °C for three days, it retained excellent tensile properties. As shown in Figure 4h, after 48 h at −25 °C, the control specimen (PS/PPy) completely froze and lost its ductility, whereas the E/PS/PA-PPy hydrogel retained good flexibility and stretchability, confirming the reliability of the synergistic antifreezing strategy under extreme low-temperature conditions.

**Figure 5:**
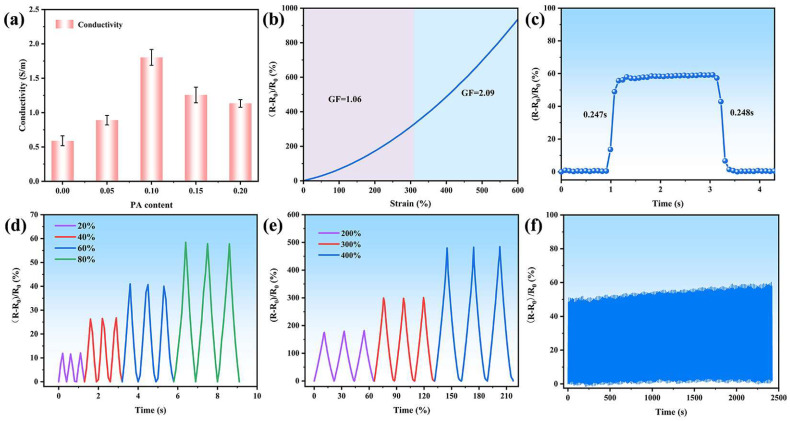
(**a**) Electrical conductivity of hydrogel as a function of PA content. (**b**) Strain–resistance response and sensitivity factor. (**c**) Single–cycle response and response/recovery time. (**d**) Real-time response under small strain (20–80%). (**e**) Real-time response under large strain (200–400%). (**f**) Cyclic stability test.

To investigate the doping effect of PA on PPy and its impact on electrical conductivity, the electrical conductivity of hydrogel samples with different PA concentrations was measured. As shown in Figure 5a, the electrical conductivity of the PS/PPy hydrogel without PA was relatively low (~0.61 S/m). As the PA concentration increased, the electrical conductivity initially rose and then declined. As shown in the electrochemical impedance spectra in Figure S2, the undoped PS/PPy hydrogel exhibits the largest ohmic internal resistance (Rs). With the increase in PA doping content, the Rs decreases significantly, and the PS/PA_0.1_-PPy sample presents the minimum Rs. Mechanistically, as a macromolecular protonic acid dopant, PA can effectively proton-dope PPy, introduce defects into the conjugated π-system and optimize the electronic structure, while providing a large number of mobile proton carriers to facilitate carrier migration. Meanwhile, PA can improve the interfacial compatibility between the PS hydrogel and PPy conductive network through hydrogen bonding, weakening the interfacial scattering loss during carrier transport. When the PA doping content further increased to 0.2, excessive PA easily induced severe aggregation of PPy molecular segments, which destroyed the continuity of the conductive network and thus was unfavorable to charge transport. This trend in conductivity corresponded to the regulation of the cross-linking structure by PA (observed in the mechanical property test results), further confirming the contribution of PA to the synergistic effect on the electro-mechanical properties of the hydrogel. When the PA doping concentration was 0.1 M, uniformly interlaced conductive pathways were formed within the hydrogel, yielding a conductivity of 1.8 S/m.

Current phytic acid-based conductive hydrogels face a fundamental limitation in the simultaneous realization of high conductivity, excellent freezing resistance and superior stretchability. The as-fabricated PS/PA-PPy hydrogel achieves an ultrahigh elongation at break of 2595.7% owing to the dynamic energy-dissipation effect of hydrophobic association microdomains. The synergistic effect of phytic acid proton doping and porous network structure endows the hydrogel with a high conductivity of 1.8 S/m. Meanwhile, phytic acid with abundant hydrogen-bonding sites can inhibit ice crystal growth and remarkably enhance the low-temperature tolerance of the hydrogel. As shown in Table 1, the PS/PA-PPy hydrogel exhibits superior comprehensive performance to previously reported phytic acid-based systems, offering a novel strategy for the design and development of high-performance flexible conductive hydrogels.

The hydrogel exhibited both excellent tensile properties and electrical conductivity, making it highly suitable for the development of flexible strain sensors. Sensitivity testing of the sensor (Figure 5b) revealed a distinct two-stage variation in the strain sensitivity coefficient, or the gauge factor. Within a wide strain range of 0–600%, the sensitivity coefficient gradually increased and reached a maximum value of 2.09, demonstrating excellent strain response sensitivity. The response and recovery times of the sensor were low, at 0.247 s and 0.248 s, respectively (Figure 5c), demonstrating its rapid strain sensing and recovery capabilities. Cyclic loading and unloading tests under different strain gradients were performed. As shown in Figure 5d, the resistive response signal remained stable under various strain conditions, with no significant signal drift or attenuation, revealing reliable cyclic response characteristics. The sensing mechanism can be inferred as follows. The hydrophobic association microdomains and dynamic hydrogen bonds realized reversible energy dissipation, dissociation and reconstruction, which synergistically enhanced the mechanical strength and self-recovery performance of the material. Proton doping of PPy with PA introduced abundant polaron carriers, and the good interfacial compatibility ensured stable charge transport upon repeated stretching. In the initial state, PPy was uniformly dispersed within the hydrogel to construct continuous conductive pathways, and the porous structure facilitated ion migration, leading to a low initial resistance. During stretching, the PPy conductive network underwent orientational separation with reduced connectivity, and the shrinkage of the hydrogel cross-section restrained ion transport, which made the relative resistance increase regularly with the applied strain.

In addition, tensile cyclic sensing tests were conducted on the hydrogel after multiple freeze–thaw cycles (Figure S3). Throughout the cycles, the baseline of (R-R_0_)/R_0_ remained stable, confirming that the hydrogel possessed reliable sensing performance and excellent low-temperature cyclic stability after freeze–thaw treatment. Multiple cyclic tensile tests (Figure 5f) further confirmed that the relative resistance changes were low, producing a stable output. Thus, the hydrogel-based strain sensor exhibited a wide detection range, high sensitivity, fast response, and excellent cyclic stability, making it highly suitable for practical industrial applications in fields such as human motion detection and smart wearable devices. As shown in Table 2, the performance of the E/PS/PA-PPy hydrogel prepared in this work was compared with that of previously reported hydrogels. The comparative results reveal that the E/PS/PA-PPy hydrogel possesses outstanding sensing performance.

**Figure 6:**
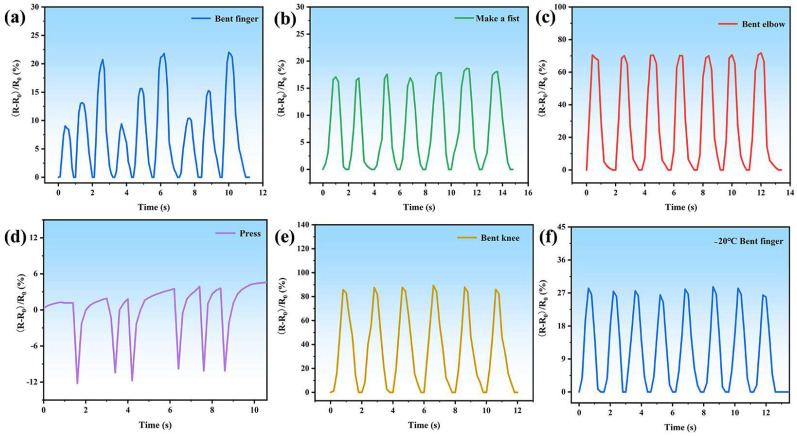
Motion detection and low-temperature sensing performance of the hydrogel sensor. (**a**) Finger bending response. (**b**) Fist-clenching response. (**c**) Elbow-bending response. (**d**) Pressure response. (**e**) Knee-bending response. (**f**) Finger−bending response at −20 °C.

Leveraging the excellent sensing performance of the hydrogel, we used it to precisely detect various human movements by monitoring changes in relative resistance in real time. As shown in Figure 6, while detecting subtle movements, the sensor produced a graded electrical signal based on the angle of finger flexion, which enabled the precise differentiation of minute limb movements. For movements of large joints such as the elbow and knee, the amplitude of the response signal increased significantly, demonstrating the wide dynamic range functionality of the sensor (Figure 6). As revealed by the compression tests, the hydrogel-based sensor effectively detected tensile deformation as well as sensitively identified changes in relative resistance caused by compressive deformation. Notably, even at −20 °C, the response signal of the sensor to finger bending was highly regular and stable, demonstrating excellent low-temperature resistance and cyclic stability. As shown in Figure S4, tensile sensing tests on the hydrogel sensor after low-temperature storage for 5 days confirm that it can still generate stable and regular sensing response signals. These characteristics collectively endow the hydrogel sensor with broad practical application prospects in wearable health monitoring and human–machine interaction devices operating in extreme environments.

## 4. Conclusions

In this study, an E/PS/PA-PPy antifreeze conductive hydrogel doped with PA was synthesized. Moreover, the effects of the preparation process and PA concentration on the microstructure, mechanical properties, antifreeze performance, and sensing capabilities of the hydrogel were systematically investigated. The results indicated that the introduction of PA optimized the PPy conductive network, regularized the microstructure, and inhibited ice crystal growth. EG and PA together formed a dual antifreezing system, effectively overcoming the limitations of conventional conductive hydrogels, such as their low-temperature freezing and performance degradation. The optimized hydrogel exhibited excellent comprehensive performance. Notably, the assembled sensor demonstrated a wide measurement range, high sensitivity, rapid response, and stable cyclic characteristics, enabling precise monitoring of human movements at both ambient and low temperatures. This study provides a feasible approach for the development of high-performance, freeze-resistant, and flexible conductive hydrogels. The materials hold significant application value and extensive potential in the fields of wearable electronics, sports rehabilitation monitoring, and human–machine interactions in extreme environments.

## Figures and Tables

**Table 1:** This work compares the performance of this PA-based hydrogel with those reported in other studies.

Component	Tensile Strain	Anti-Freezing	Conductivity	Ref.
PAM/CMC/LMA/PA	1476%	−25°C	1.45 S/m	[34]
PLM (PVA, LM@PA)	~620%	×	0.53 S/m	[35]
HAPAM/PA@Fe^3+^	1299%	−31°C	1.2 S/m	[36]
PA-PAAM-CS	760.9%	−47.3%	~1.2 S/m	[37]
PS/PA-PPy	2595.7%	−17.4°C	1.8 S/m	Our work

**Table 2:** The performance comparison of this E/PS/PA-PPy hydrogel with other reported hydrogels.

Component	Anti-Freezing	Strain Sensors		Response Time	Ref.
		Detection Strain Range	Gauge Factors		
PAM/CMC/LMA/PA	−25°C	1–500%	1.12	~0.4 s	[34]
HBP/CNF-PA	−40°C	0–120	0.81	~0.29 s	[38]
		120–300	1.15		
		300–600	1.34		
PVA-SbQ/CNC-PPy/APS/FeCl_3_	^___^	0–150%	1.02	~0.46 s	[39]
		150–420%	1.43		
PVA/EG	−20°C	0–100%	0.725	^___^	[40]
E/PS/PA-PPy	−42.3°C	0–300%	1.06	~0.25 s	Our work
		300–600%	2.09		

## Data Availability

The datasets used and analyzed in the current study are available from the corresponding author on reasonable request.

## Supplementary Materials

The following supporting information can be downloaded at: https://www.mdpi.com/article/10.3390/polym18111299/s1, Table S1: The detailed compositions of hydrogels; Figure S1: TG curves of PS/PPy hydrogel and PS/PA_0.1_-PPy hydrogel; Figure S2: Electrochemical impedance spectra of PS/PPy and PS/PA-PPy hydrogels with different PA doping concentrations; Figure S3: Relative resistance change of the hydrogel during repeated tensile cycles after multiple freeze-thaw treatments; Figure S4: Cyclic tensile sensing response curves of E/PS/PA-PPy hydrogel sensor after being stored at −20 °C for 5 days via manual stretching test.
