# Supplementary material for: Design of Conductive Hydrogels Based on the Synergistic Effects of Hydrophobic Frameworks and Dual Antifreeze Strategies, Suitable for Wearable Flexible Sensors
Source: Polymers (Basel). 2026 May 25;18(11):1299. doi: 10.3390/polym18111299 (PMC13259541; doi:10.3390/polym18111299)
Supplement: Supplementary file 1 [file polymers-18-01299-s001.zip › polymers-4267245-supplementary.pdf]

Supplemental Information

Design of Conductive Hydrogels Based on the Synergistic Effects of Hydrophobic Frameworks and Dual Antifreeze Strategies, Suitable for Wearable Flexible Sensors

Jijun Luo [1,2], Sainan Wang [1,3], Xiangtong Jian [4], Kenan Yang [3,4], Bin Du [3,4], Mengwei Yin [4] and Shisheng Zhou [3,4,*]

[1] School of Mechanical Engineering, Xi'an University of Technology, Xi'an 710054, China
[2] Shaanxi Energy Institute, Xianyang 712000, China
[3] Shaanxi Collaborative Innovation Center of Green Intelligent Printing and Packaging, Xi'an University of Technology, Xi'an 710054, China
[4] Faculty of Printing, Packaging Engineering and Digital Media Technology, Xi'an University of Technology, Xi'an 710048, China
* Correspondence: zhoushisheng@xaut.edu.cn

Table S1. The detailed compositions of hydrogels.

Sample	AA (g)	SMA (g)	CTAB(g)	APS(g)	PA(mol/L)	Py(ml)
PS	10	1	3	0.1	0	0
PS/PPy	10	1	3	0.1	0	0.55
PS/PA$_{0.05}$-PPy	10	1	3	0.1	0.05	0.55
PS/PA$_{0.1}$-PPy	10	1	3	0.1	0.1	0.55
PS/PA$_{0.15}$-PPy	10	1	3	0.1	0.15	0.55
PS/PA$_{0.2}$-PPy	10	1	3	0.1	0.2	0.55

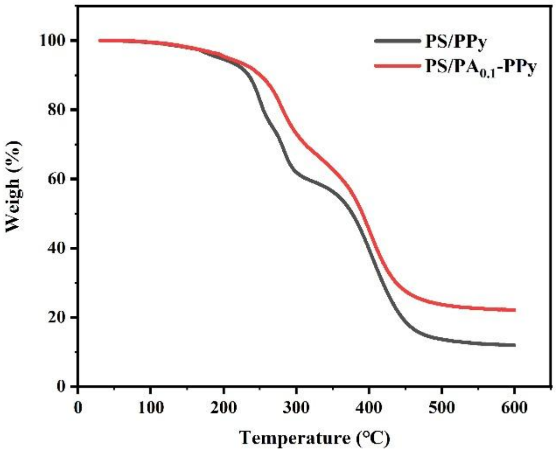

Figure S1. TG curves of PS/PPy hydrogel and PS/PA$_{0.1}$-PPy hydrogel.

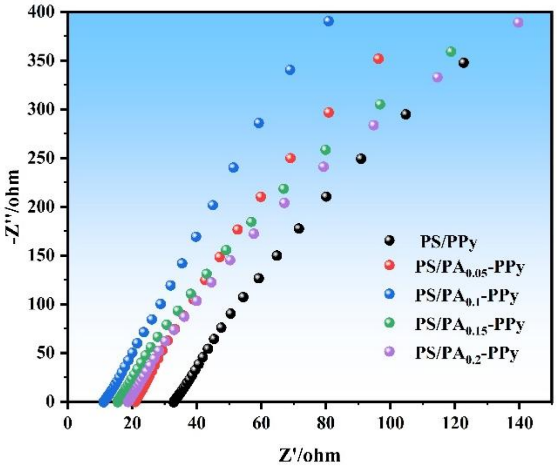

Figure S2. Electrochemical impedance spectra of PS/PPy and PS/PA-PPy hydrogels with different PA doping concentrations.

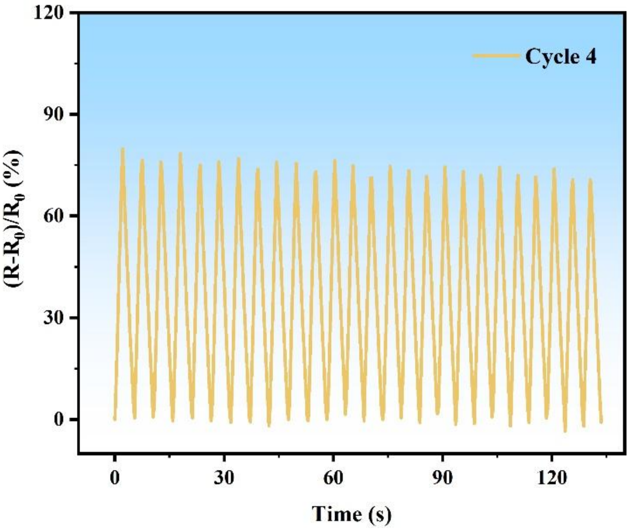

Figure S3. Relative resistance change of the hydrogel during repeated tensile cycles after multiple freeze-thaw treatments.

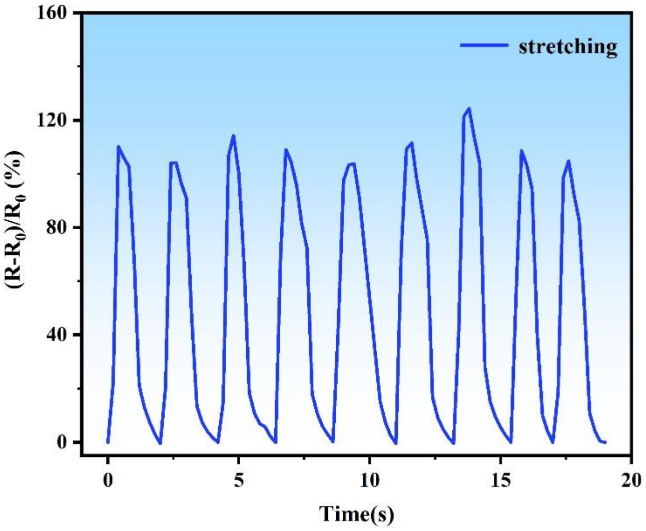

Figure S4. Cyclic tensile sensing response curves of E/PS/PA-PPy hydrogel sensor after being stored at −20 °C for 5 days via manual stretching test.
